# The TRIXS end-station for femtosecond time-resolved resonant inelastic x-ray scattering experiments at the soft x-ray free-electron laser FLASH

**DOI:** 10.1063/4.0000029

**Published:** 2020-09-16

**Authors:** S. Dziarzhytski, M. Biednov, B. Dicke, A. Wang, P. S. Miedema, R. Y. Engel, J. O. Schunck, H. Redlin, H. Weigelt, F. Siewert, C. Behrens, M. Sinha, A. Schulte, B. Grimm-Lebsanft, S. G. Chiuzbăian, W. Wurth, M. Beye, M. Rübhausen, G. Brenner

**Affiliations:** 1 DESY, Notkestr. 85, Hamburg 22607, Germany; 2 European XFEL GmbH, Holzkoppel 4, 22869 Schenefeld, Germany; 3 Institute of Nanostructure and Solid State Physics, University of Hamburg and Center for Free-Electron Laser Science (CFEL), Notkestr. 85, Hamburg 22607, Germany; 4 Sorbonne Université, CNRS (UMR 7614), Laboratoire de Chimie Physique-Matière et Rayonnement, 4 Place Jussieu, 75252 Paris Cedex 05, France; 5 Physics Department, Universität Hamburg, Luruper Chaussee 149, 22761 Hamburg, Germany; 6 Helmholtz Zentrum Berlin, Department Optics and Beamlines, Albert-Einstein-Strasse 15, 12489 Berlin, Germany

## Abstract

We present the experimental end-station TRIXS dedicated to time-resolved soft x-ray resonant inelastic x-ray scattering (RIXS) experiments on solid samples at the free-electron laser FLASH. Using monochromatized ultrashort femtosecond XUV/soft x-ray photon pulses in combination with a synchronized optical laser in a pump-probe scheme, the TRIXS setup allows measuring sub-picosecond time-resolved high-resolution RIXS spectra in the energy range from 35 eV to 210 eV, thus spanning the M-edge (M_1_ and M_2,3_) absorption resonances of 3d transition metals and N_4,5_-edges of rare earth elements. A Kirkpatrick–Baez refocusing mirror system at the first branch of the plane grating monochromator beamline (PG1) provides a focus of (6 × 6) *μ*m^2^ (FWHM) at the sample. The RIXS spectrometer reaches an energy resolution of 35–160 meV over the entire spectral range. The optical laser system based on a chirped pulse optical parametric amplifier provides approximately 100 fs (FWHM) long photon pulses at the fundamental wavelength of 800 nm and a fluence of 120 mJ/cm^2^ at a sample for optical pump-XUV probe measurements. Furthermore, optical frequency conversion enables experiments at 400 nm or 267 nm with a fluence of 80 and 30 mJ/cm^2^, respectively. Some of the first (pump-probe) RIXS spectra measured with this setup are shown. The measured time resolution for time-resolved RIXS measurements has been characterized as 287 fs (FWHM) for the used energy resolution.

## I. INTRODUCTION

High-resolution resonant inelastic x-ray scattering in the soft x-ray range has developed into a powerful technique for studying low-energy excitations in condensed matter, and probing electronic, orbital, spin, and lattice degrees of freedom in an element-specific and chemical selective manner (Ament *et al.*, 2011). Of particular interest are correlated materials with a complex interplay of these degrees of freedom or catalysts with their clear importance for applied sciences and life. Furthermore, combining time-resolved techniques with RIXS offers the opportunity to study the ultrafast dynamics of quasi-particles, e.g., phonons and magnons, when a material is photo-excited into a transient state and then probed by an x-ray pulse. With the advent of free-electron lasers (FELs) providing ultrashort, coherent, and highly brilliant photon pulses, timescales in the sub-ps and few-fs range can be accessed by a large variety of time-resolved pump-probe methods. However, because of small inelastic scattering cross sections even for resonant excitation, RIXS is still one of the most photon-hungry methods. Successful experiments thus require combining the most advanced photon sources with dedicated instrumentation for time-resolved studies. The setup should have a sufficient time- and energy resolution, while the throughput of the spectrometer should be maximized. The high-repetition rate free-electron laser FLASH at DESY in Hamburg provides typically 5000 ultrashort soft x-ray pulses per second of 10–100 fs (FWHM) duration in the fundamental energy range from 30–300 eV, thus covering most of the M- and N-edges of transition metals and rare-earth elements, respectively (Ackermann *et al.*, 2007).

Utilizing RIXS within a pump-probe scheme—either optical pump x-ray probe or all x-ray pump-probe—makes it a very powerful technique to study the dynamics of involved states and their interactions. Such experiments have already been performed in the past at FLASH (Beye *et al.*, 2010), at the Linac Coherent Light Source (LCLS) (Wernet *et al.*, 2015, Dell'Angela *et al.*, 2013 and Dean *et al.*, 2016) as well as at the Center for Free-Electron Laser Radiation for Multidisciplinary Investigations (FERMI) (Dell'Angela *et al.*, 2016) and recently again at the LCLS (Parchenko *et al.*, 2020) on different systems ranging from strongly correlated materials to catalysts. Beye *et al.* clearly demonstrated the first-order nature of the liquid–liquid phase transition in silicon via time-resolved soft x-ray emission spectroscopy. The authors used femtosecond optical laser pulses at 400 nm to drive the phase transition in silicon and probed it via soft x-ray emission spectroscopy utilizing femtosecond FEL pulses at 117 eV (Beye *et al.*, 2010). Wernet *et al.* performed orbital-specific mapping of the ligand exchange dynamics of Fe(CO)_5_ in solution at LCLS. In the experiment, the valence electrons were pumped with a 266 nm femtosecond laser and the removal of CO was observed via Fe L_3_-edge RIXS at 711.5 eV. Sub-picosecond dynamics of the photo-induced ligand detachment has been observed and the electronic structure of the photo fragments has been characterized (Wernet *et al.*, 2015). Dell'Angela *et al.* have used a commercial RIXS spectrometer at FERMI and measured for the first time high-resolution EUV RIXS at a seeded FEL on a KCoF_3_ single crystal at Co M_2,3_-edge (62 eV). The instrument demonstrated 120 meV resolution and has great potential for high time-resolution RIXS studies (Dell'Angela *et al.*, 2016). Dean *et al.* studied the ultrafast photo-induced magnetic dynamics in Sr_2_IrO_4_ perovskite and discovered significantly different time scales for 2D and 3D antiferromagnetic ordering in the system on the order of a few picoseconds and few hundreds of picoseconds, correspondingly. Here, the sample was pumped by a 2 *μ*m wavelength NIR laser pulse and probed via RIXS at the Ir L_3_-edge at approximately 11.2 keV (Dean *et al.*, 2016). Very recently, Parchenko *et al.* demonstrated a sub-picosecond orbital dynamics during a photoinduced insulator to metal phase transition in V_2_O_3_, a classical Mott-Hubbard material by the time-resolved RIXS at Vanadium L_2,3_-edge (523 eV) performed at the soft x-ray materials science (SXR) beamline (Parchenko *et al.*, 2020). These experiments with energy and temporal resolution already achieved have clearly demonstrated the enormous potential of the time-resolved RIXS method and motivate its further development.

In this paper, we report on the dedicated experimental end-station TRIXS for femtosecond time-resolved (tr-) high energy resolution RIXS on solid samples, located at the PG1 monochromator beamline branch (Dziarzhytski *et al.*, 2016) at FLASH. The combination of the ultra-short and highly brilliant photon pulses of FLASH and its high repetition rate of up to 5 kHz (Tiedtke *et al.*, 2009) is well suited for time-resolved soft x-ray spectroscopy experiments of different kinds, which are routinely performed in a reasonable measurement time at the facility (Pontius *et al.*, 2018, Schmid *et al.*, 2019, Erk *et al.*, 2018, Zapolnova *et al.*, 2020, Kutnyakhov *et al.*, 2020).

## II. THE EXPERIMENTAL SETUP

### A. Overview

**FIG. 1.**
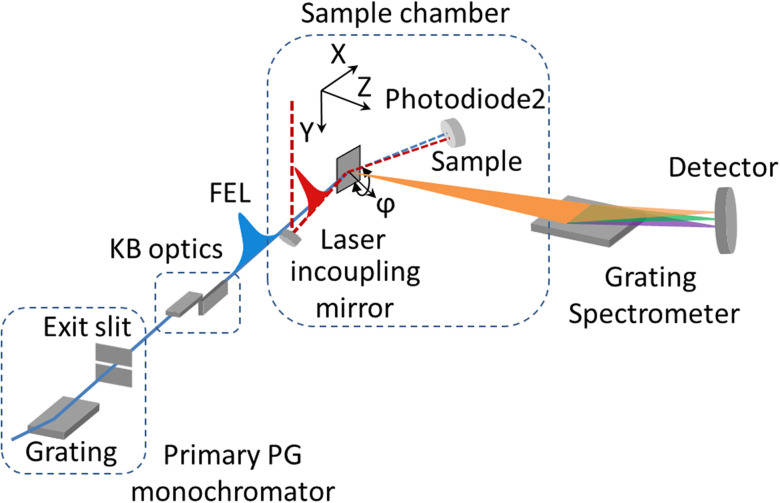
Schematic overview of the TRIXS end-station including the monochromator beamline PG1 and its focusing optics. Spectrometer is shown without focusing optics.

The TRIXS end-station is installed at the PG1 beamline branch (Dziarzhytski *et al.*, 2016) of the FLASH1 plane grating (PG) monochromator beamline (Martins *et al.* 2006, Wellhöfer *et al.* 2007, Gerasimova *et al.*, 2011) which provides a photon energy bandwidth down to a few meV and a flux at the sample on the order of 10^10^ photons per pulse at the fundamental wavelength of the FEL. The main components of the setup are the sample chamber, the RIXS grating spectrometer, and the optical laser system (see Fig. 1) (Biednov *et al.*, 2019). The monochromatized FEL beam is focused onto the sample by a Kirkpatrick-Baez (KB) mirror system (Kirkpatrick and Baez, 1948, Siewert *et al.*, 2010, Dziarzhytski *et al.*, 2016). The elastically and inelastically scattered photons are collected and energy-resolved by the spectrometer. For tr-RIXS experiments, the optical laser is coupled into the ultra-high vacuum (UHV) sample chamber in a quasi-collinear fashion. Tools and diagnostics to establish spatial and temporal overlap are available and described in Secs. II B and II F.

### B. The sample chamber

**FIG. 2.**
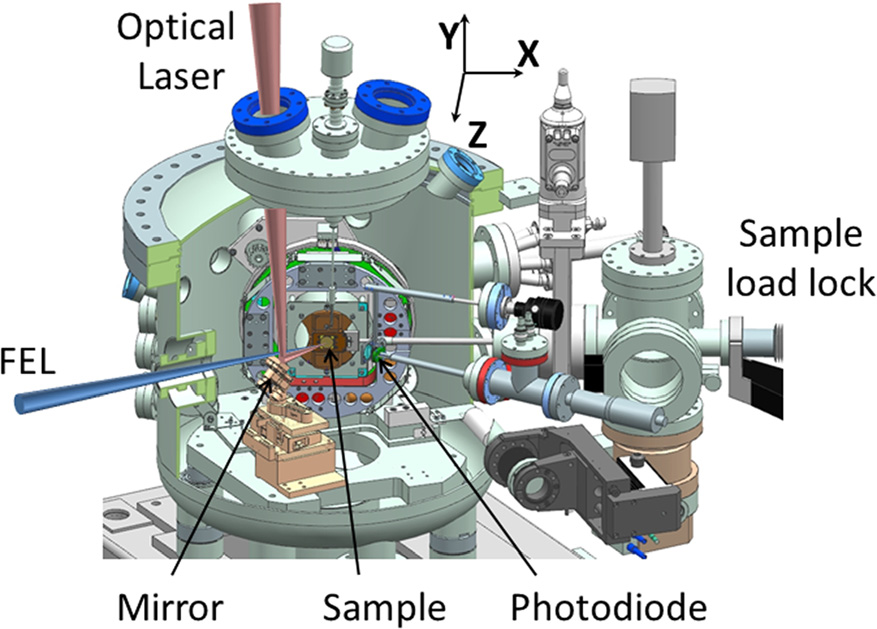
Schematic view of the TRIXS sample chamber. The focused FEL beam impinges on the sample at 30° incident angle relative to its surface. The optical laser beam enters the chamber from the top and is directed to the sample by the mirror. Directly reflected beams are detected by a photodiode which is a part of the “time zero” diagnostics described later. A sample load lock system is used to exchange samples during the beamtime without venting the sample chamber. The Z-axis points toward the TRIXS spectrometer, not shown here; X and Y axes are directed along the sample surface.

The sample chamber is schematically shown in Fig. 2. It is designed for solid samples and is operated under UHV working conditions with pressures of typically 10^−9^ mbar.

The sample itself is mounted on a goniometer manipulator which has four degrees of freedom for precise sample adjustment, namely movements along the horizontal X- and Z-axis, the vertical Y-axis and azimuthal rotation around the sample surface normal (angle *ϕ* in Fig. 1). The translational adjustments and *ϕ*-rotation can be done with an accuracy of 1 *μ*m and 0.1°, respectively. These precise settings are crucial for placing the sample into the overlapping foci of the beamline and spectrometer (see also Sec. II C). Note, that the Z-axis coincides with the spectrometer optical axis and is fixed at 80° relative to the X-axis and normal to the Y-axis. The angle of incidence on the sample is 30° relative to its surface and can be altered by using wedged sample holder inlets. The azimuthal rotation *ϕ* changes the relative orientation of the X- and Y-axis in the lab frame; the movement in the XY plane enables sample surface scans. Furthermore, the Z-movement allows one to compensate for different sample thicknesses, aligning the sample surface to the beam path.

Samples are attached to a sample holder inlet. Its dimension is (20 × 20) mm^2^ and the sample can be exchanged via a load lock system without breaking the vacuum of the main chamber. Besides the samples of interest, the sample holder always hosts a Ce:YAG polished crystal fixed on top of a reference pinhole in order to control the spatial overlap of the FEL and the optical laser by observing the fluorescence generated by the crystal. The spatial overlap is monitored by a microscope camera with 10 *μ*m spatial resolution. Furthermore, either a Si_3_N_4_ film on a Si substrate or a GaAs crystal is installed as part of the time zero tool to establish the temporal overlap between the FEL and optical laser pulses by observing changes of the optical reflectivity induced by the absorption of the XUV pulse. The working principle of the time zero tool will be described in more detail below (see Sec. II F). An in-vacuum dielectric coated in-coupling mirror with low group delay dispersion (GDD) provides quasi-collinear in-coupling of the optical laser. Last but not least, the sample chamber is equipped with a flow cryostat using liquid ^4^He which is, in the present assembly, able to control the sample temperature between 65 K and 800 K.

### C. The spectrometer layout

**FIG. 3.**
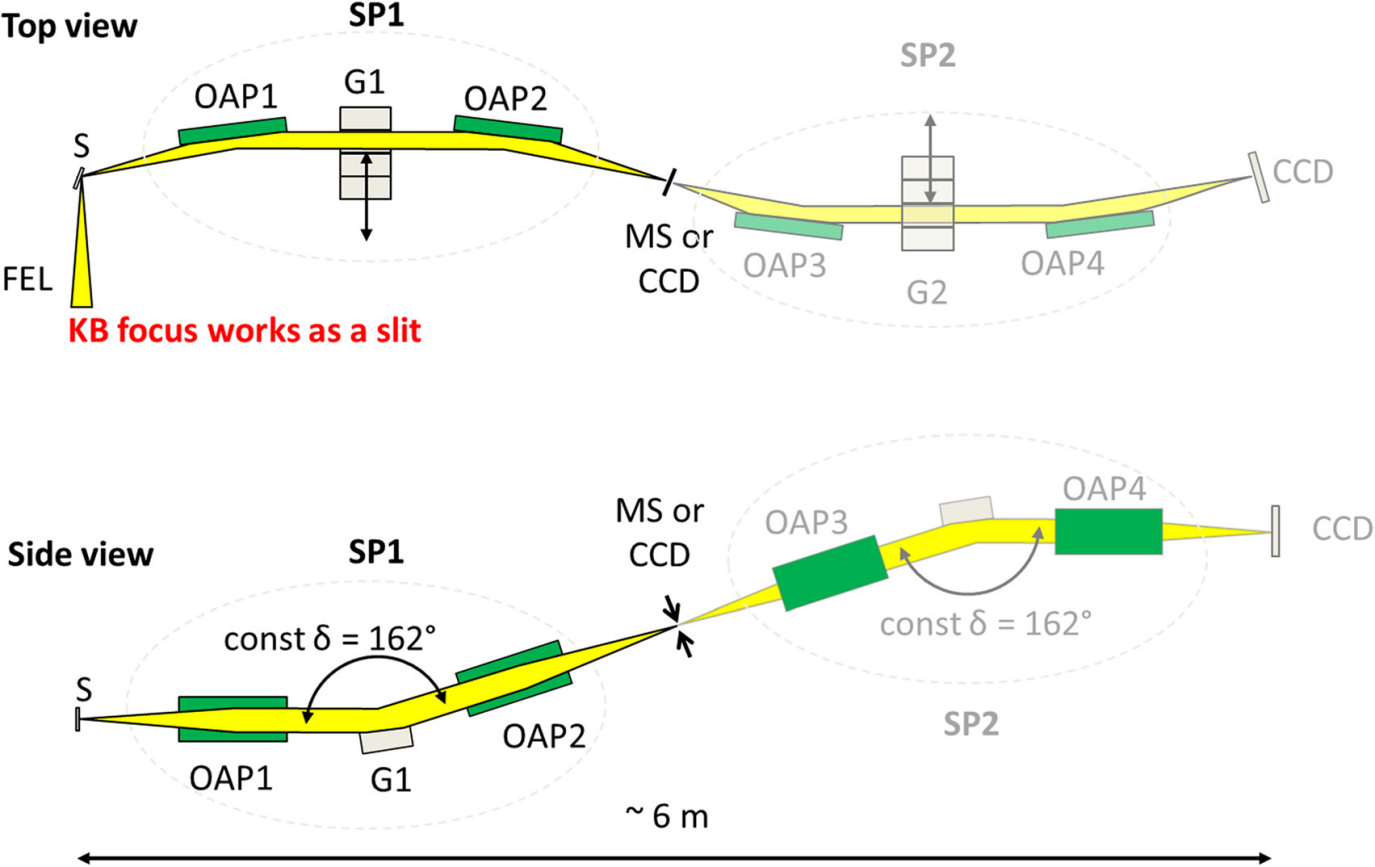
Optical layout of the TRIXS spectrometer. SP1 is the collecting and first dispersive stage, while SP2 is the second spectrometer stage. S is the sample; G1/2—are grating units hosting up to 4 gratings; MS is the middle slit; and CCD is a charge-coupled device camera. The optical laser in-coupling is not shown here. In the current configuration, the MS is replaced by a CCD camera and only SP1 is used as the spectrometer within TRIXS.

The design and development of the high-resolution soft x-ray RIXS spectrometer has been described in (Rübhausen *et al.*, 2004, Rusydi *et al.*, 2014 and Biednov *et al.*, 2019) and is shown in Fig. 3. Many RIXS spectrometers/beamlines all over the world optimized for shorter wavelengths (MERIXS at the Advanced Light Source (Chuang *et al.*, 2012), ADRESS at the Swiss Light Source (Strocov *et al.*, 2010), the commercial Scienta XES 355 RIXS spectrometer at FERMI FEL (Dell'Angela *et al.*, 2016), and AERHA at Soleil (Chiuzbăian *et al.* 2014, etc.), are based on a scheme with a variable line spacing grating, sometimes in combination with an additional focusing mirror. In contrast, TRIXS utilizes a Czerny–Turner optical concept (Czerny and Turner, 1930) which is often implemented in UV/VIS Raman spectrometers (Schulz *et al.*, 2005). Briefly, TRIXS is an imaging grating spectrometer which consists of two confocal coupled monochromator stages, designed to cover the spectral range from 35 to 210 eV, intended to provide a spectral resolution of 2–15 meV and efficient elastic line suppression to allow for the study of low energy excitations in solid samples. Both spectrometer stages employ blazed plane diffraction gratings (Dziarzhytski *et al.*, 2018) working in inside and outside orders, respectively, at a constant included angle *δ* of 162°. The first stage acts as an entrance objective that collects a relatively large solid angle and reduces the stray-light by several orders of magnitude by dispersing the light, thus separating the elastic and inelastic contributions. A middle slit placed in between the monochromators blocks the elastic contribution and allows only inelastically scattered photons to propagate into the second high-resolution monochromator stage. The second stage acts as the spectrograph which disperses the light further and focuses it onto the detector plane. Off-axis parabolic (OAP) mirrors are used in grazing incidence for collimating and refocusing as shown in Fig. 3. The magnification factor of the spectrometer in 0th order is 2.18. All optical elements are coated with amorphous diamond-like carbon (DLC) of 45 nm thickness (Störmer *et al.*, 2008). Table I summarizes the specifications of all optical components of the first spectrometer stage. The spectrometer is kept under UHV at a pressure on the order of 10^−8^ mbar.

**TABLE I.** Specifications of the optical elements of the first stage of the TRIXS spectrometer.

Element	Form factor	W × L, mm	Focal length, m	Slope error, arc sec rms	Micro rough., nm rms	Spectral range, eV/acceptance angle, mrad	Incidence/blaze (B) angle, °
Collimat. mirror	Off-axis parabola	20 × 390	0.55	Mer. < 0.75 Sag. < 0.96	0.44	H × V = 82.6 × 37	83
Grating 576 l/mm	Plane, constant ruling	54 × 175	…	Mer. < 0.06 Sag. < 0.1	Groove <1	36–210 (first and second orders)	1.82(B)
Focusing mirror	Off-axis parabola	45 × 398	1.2	Mer. < 0.8 Sag. < 1	0.85	…	83

As mentioned above, the KB mirrors focus the FEL beam onto the sample. In fact, they image the vertical PG1 exit slit onto the sample position (demagnification factor of 4.9), while the horizontal beamline focus, which lies one meter upstream, is demagnified by a factor of 9.9. Thus, the vertical focus size at the sample can be adjusted by opening or closing the PG1 beamline exit slit. Operating the primary PG1 beamline monochromator in the high-resolution mode with 20 *μ*m exit slit size, the focus spot size at the sample position was measured to be (6 × 6) *μ*m^2^ FWHM (Dziarzhytski *et al.*, 2016). The spectrometer does not use an entrance slit, so the vertical size of the illuminated spot on the sample enters the spectrometer resolution. Its contribution can be calculated using the following formula: *Δλ* = *F·*cos(*α*)/(*k·m·R*), where *Δλ* is the spectral resolution limit due to the vertical spot size (F) at the sample, *α* is the incident angle on the grating, k is the spectrometer grating groove density, *m* is the diffraction order (m = 1, 2), and *R* is the distance from the sample to the first mirror (OAP1 in Fig. 3). The vertical spot size on the sample, together with the width of the middle and exit slits, dominates the resolution of the spectrometer compared to the influence of slope errors of the mirrors and the gratings. The light scattered from the sample is collimated by OAP1 and directed onto the grating unit G1 (see Fig. 3), which works in an inside order configuration. Note, that the parabolic mirrors deflect the beam perpendicular to the dispersion plane. The maximum acceptance angle of light scattered from the sample to OAP1 (and therefore the acceptance angle of the whole spectrometer) is (37 × 82.6) mrad^2^ (V × H), which is substantially larger than realized in most of the spectrometers that are optimized for shorter wavelengths (Chuang *et al.*, 2012, Chiuzbăian *et al.*, 2014, Beye *et al.*, 2019). The dispersed photons are collected and focused onto the middle slit by the second mirror OAP2.

Due to the very low tolerances of parabolic mirrors, the precise alignment of the spectrometer is critical (the acceptable spectrometer misalignment relative to the source is < 20 *μ*m in X, Y, and Z directions). Optical interferometry and wavefront sensing techniques have been applied to guarantee the operation of the first spectrometer stage within the designed specifications and are described in detail in (Dziarzhytski *et al.*, 2018, Biednov *et al.*, 2019). In the current baseline configuration, the first stage is fully operational. It reaches an energy resolution of 35–160 meV over the entire spectral range and is being used for user experiments since December 2018. Since the second monochromator stage is currently not used, the middle slit system is replaced by a CCD camera.

### D. The detection scheme

In order to record the RIXS spectra, a back-illuminated CCD camera [Princeton PIXIS-XO (512 × 2048) pixels, pixel size 13.5 *μ*m] is installed at the spectrometer middle slit position. The camera chip is cooled to −70 °C to minimize thermal noise and is triggered by the PG1 beamline fast shutter (Plönjes and Tiedtke, 2015) which is operated in a special chopper mode, having N seconds opening time (exposure) and M seconds closing time (for readout), N ≫ M.

The optical laser used for tr-RIXS experiment is scattered from the sample and can produce a substantial background signal on the spectrometer camera. To block stray light mainly from the optical laser and other possible sources, an exchangeable thin film filter, usually 100 nm aluminum or zirconium, is implemented in front of the in-vacuum CCD detector.

In general, the low quantum yield of the RIXS process, as well as the limited acceptance and throughput of spectrometers, detection efficiency, and signal processing, result in rather low signal-to-noise (S/N) ratios. Especially challenging is the here used XUV spectral range, where the single-photon signal is below the typical CCD readout noise. This prevents the algorithmic discrimination between noise and photon signals as it is often used for higher photon energies (Amorese *et al.*, 2014). In order to minimize the influence of the detector readout and ADC quantization noise—which are the limiting factors in RIXS experiments with very low signals—the lowest pixel reading frequency of 100 kHz is used. The exposure time of the CCD camera is typically increased to several seconds, in order to increase the integral signal above the readout noise level. However, this approach comes at the expense of energy-, and time-resolution due to the accumulation over several FEL/optical laser bursts, thus also integrating over the timing and pointing jitter of both sources. As will be discussed in detail in Sec. II F, the major contribution to the temporal resolution is the convoluted FEL and optical laser pulse durations. The integration over the pointing jitter mostly affects the energy resolution of the TRIXS spectrometer, as it varies the spectral bandwidth through the PG monochromator exit slit. Pointing instability also affects the FEL focus position on the sample, which results in an effectively larger illuminated spot (smearing effect), thus reducing the spectral resolution of the spectrometer when integrating the signal over many shots.

We want to note, that alternative detection schemes with faster readout have in principal the potential to improve the effective energy and time resolution of the TRIXS instrument. Unfortunately, silicon-based direct electronic detectors (like CCDs or CMOS chips) are challenging to fabricate with a high quantum efficiency in the required photon energy range, high spatial resolution, high readout speed, and readout noise substantially below the single-photon signal. Such detectors are currently not available and the used one provides a viable compromise. Alternative schemes based on multi-channel plate (MCP) amplifiers suffer from a lower quantum efficiency, lower spatial resolution, and strongly reduced dynamic range (i.e., they easily saturate in the elastic line). Further developments of other detection schemes are required.

### E. The optical pump-probe laser

The FLASH Ti:Sa femtosecond “burst-mode” optical laser has an optical parametric chirped pulse amplifier and provides a fundamental central wavelength tunable in the range from 770 to 830 nm with variable pulse duration between 90 fs and 1 ps FWHM. Typical pulse energies reached in the fundamental are around 30 *μ*J (Redlin *et al.*, 2011). The laser oscillator is optically synchronized to the FLASH master laser oscillator which minimizes the jitter down to 10–20 fs (rms) (Schulz *et al.*, 2013). The timing pattern of the optical laser with up to 400 pulses at 1 *μ*s spacing in a burst repeated ten times a second reproduces the burst timing pattern of the FEL exactly. The laser can be shared between different FLASH1 beamlines and is delivered to the end-station via low-vacuum laser transport beamlines terminated by a fused silica window. The single pulse energy after transport as measured at the beamline exit window is about 10–15 *μ*J.

**FIG. 4.**
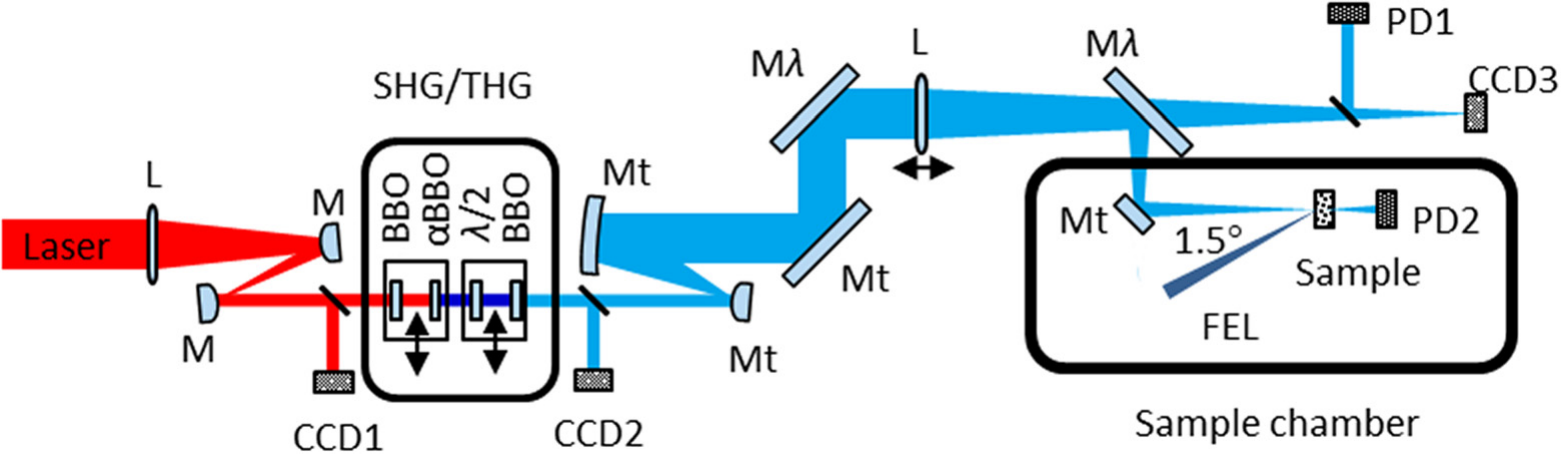
Optical scheme of the TRIXS laser setup. The laser light propagates from left to right. L denotes focusing lenses; M are 800 nm mirrors; Mt are triple-band coated mirrors for 266 nm, 400 nm, and 800 nm; Mλ are mirrors which are coated for a single wavelength, and need to be exchanged for different wavelengths; CCD1/2 are beam position monitor cameras; CCD3 is the virtual focus detector camera; and PD1/2 are photodiodes for monitoring the incoming laser intensity and for the time zero tool, correspondingly.

A frequency converter setup is used to generate the second and third harmonics (SHG/THG) of the optical laser, i.e., 400 nm and 267 nm, which allows us to cover the band gaps of almost all oxides of transition metals and rare earth elements. The laser in-coupling was designed as collinear as possible to the FEL beam (1.5° angle between beam axes) in order to keep the path length of the pump and probe beams unchanged while scanning over the sample surface, and to optimize the overall time resolution. The optical scheme of the laser transport set-up is shown in Fig. 4 and the performance parameters are listed in Table II.

**TABLE II.** Optical laser parameters at the TRIXS sample chamber.

Wavelength, nm	Focus size V × H FWHM, *μ*m^2^	Fluence, mJ/cm^2^
800	100 × 200	∼120
400	80 × 160	∼80
267	50 × 100	∼30

Two telescopes are used within the laser beam path: one for beam diameter demagnification by a factor of 20 for efficient frequency conversion; and the second one to expand the beam size by a factor of 50 before focusing onto the sample. Both telescopes are based on spherical mirrors working at inclination angles of 9°. This produces a slightly astigmatic beam. However, this aberration can be largely corrected by adjusting the distance between the mirrors. So it does not affect the frequency conversion or the focusing properties substantially. All mirror coatings are optimized for 99.9% reflectivity and lowest GDD at the wavelength of interest. The last mirror M*λ* in Fig. 4 has 98.5% reflectivity. The leakage light of that mirror is used to monitor the “virtual” laser focus as explained below. Two M*λ* mirrors are single-wavelength coated and used to filter the remaining contribution of the lower harmonics. These mirrors must be changed for the particular wavelength of choice.

The non-linear frequency converter (marked as “SHG/THG” in Fig. 4) is based on the concept described in (Enqvist, 2004) and is built as a retractable unit. It employs a 0.3 mm thick beta-barium borate (BBO) type I crystal for SHG followed by an α-BBO crystal (0.6 mm thick) to compensate for the time delay between the fundamental and second harmonic. The third harmonic is generated by the second BBO crystal (0.25 mm thick) at the end of the unit. A *λ/2* waveplate in front of the THG crystal is used to rotate the polarization of the second harmonic. For using the third harmonic of the optical pulses in an experiment, both crystals are in place. For using the second harmonic, only the SHG crystal is used.

The focused laser beam enters the sample chamber via a triple-band anti-reflection coated viewport flange of optical surface quality. The focus size at the sample can be controlled by adjusting the distance of the focusing lens L, which is mounted on a motorized linear stage between both Mλ mirrors (see Fig. 4).

The beam position and pointing are monitored by three CCD cameras installed in the near and far-field (CCD1/2 and CCD3). CCD3 is mounted at the same distance from the focusing lens L as the distance from the lens L to the sample, thus working as a virtual focus monitor. This camera uses the light leaking from the last deflection/filtering mirror M*λ*.

The laser intensity upstream and downstream of the sample is measured by two fast photodiodes (PD1/2, Model AXUV63HS1) connected to ADCs which are included into the FLASH Data Acquisition (DAQ) system (Agababyan *et al.* 2007). The in-vacuum photodiode PD2 is equipped with a retractable filter holder hosting a 0.5 mm thick fused silica plate and 100 nm thick Al or Zr filters, and is used for the time zero tool explained in Sec. II F.

### F. Time zero tool

Pump-probe experiments rely on stable spatial overlap and a well-defined time delay between the FEL and optical laser. At TRIXS, the spatial overlap of the FEL and the optical laser is controlled by monitoring the beam positions with a Ce:YAG crystal located at the sample position by using a CCD camera with a telecentric lens featuring 10 *μ*m spatial resolution. The temporal overlap between two pulses on a femtosecond timescale is established in two steps. First, the coarse time overlap with approximately 250 ps accuracy is achieved by monitoring the signal from the photodiode PD2 caused by both the FEL and laser pulses reflected from a GaAs or Si_3_N_4_ sample (see Fig. 1) on a fast digital oscilloscope.

**FIG. 5.**
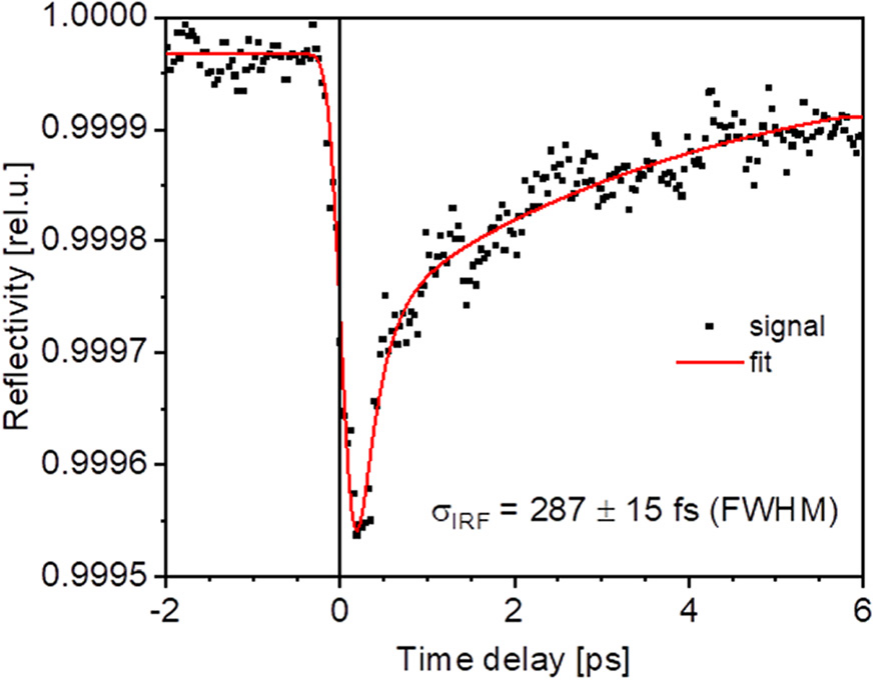
Transient optical reflectivity probed with 267 nm on Si_3_N_4_ induced by 70.4 eV photons. The FWHM of the reflectivity drop is 287 ± 15 fs and is essentially limited by the FEL pump and laser probe pulse durations. Black data points are the experimental data and the red curve is the fit. The vertical black line indicates the position taken as time zero.

In a second step, the search for the exact time overlap (fine time zero) is based on a measurement of transient reflectivity changes of the optical laser induced by the FEL as a function of the time delay (Gahl *et al.*, 2008, Beye *et al.*, 2012, Eckert *et al.*, 2015]. Briefly, the FEL pulse excites electrons into the conduction band of the Si_3_N_4_ or GaAs sample, which changes the electron and hole density across the bandgap and thus the material's optical constants. By scanning the time delay between FEL and optical laser, one can trace the intensity change of the reflected optical laser beam as a function of delay between the pulses (see Fig. 5). In order to measure these small reflectivity changes, the FEL is operated at a half intra-burst repetition rate (e.g., 500 kHz) compared to the optical laser (1 MHz), thus providing pumped and unpumped pulses in an alternating fashion within one laser burst. By using the ratio of the photodiode signal for these two intensity patterns vs delay line position of the optical laser, one can measure time zero with a precision dominantly limited by the convolution of the FEL and optical laser pulses. The instrument temporal response function (IRF) of TRIXS can be extracted from this measured transient reflectivity signal. Several factors contribute to the IRF as will be discussed in the following: the overall time response σ_IRF_ = √(σ_FEL_^2^ + σ_Las_^2^ + σ_FELtrain_^2^ + σ_LasTrain_^2^ + σ_FEL-Las_^2^) is a convolution of the effective FEL pulse duration after the PG monochromator (σ_FEL_, 120–250 fs), the optical laser pulse duration (σ_Las_, 90–120 fs), the FEL intra-train jitter (σ_FELtrain_, 30–40 fs), the optical laser intra-train jitter (σ_FELtrain_, has never been measured, but assumed here to be on the same order of magnitude as the FEL intra-train jitter), and the relative arrival time jitter of the two lasers (σ_FEL-Las_, 40–60 fs) (Schulz *et al.*, 2013). The duration of the Auger cascade that induces the change in optical constants as a result of XUV photoabsorption is not discernible on top of the other contributions and can be neglected here. All pulse durations and jitters are given in FWHM. The FEL pointing jitter has a marginal effect on temporal broadening. The FEL and laser beams are focused onto the sample in a quasi-collinear fashion, having 1.5° angle between their beam centers (see Fig. 4). At an FEL beam incidence angle on a sample of 30° relative to its surface and a focal spot size of 10–20 *μ*m, this leads to 0.2 fs elongation only. The pointing jitter changes the relative angle between the FEL and laser beam only by a fraction of a degree and is therefore not contributing to the temporal resolution significantly.

In the present example of Fig. 5, σ_IRF_ was found to be 287 ± 15 fs FWHM. A convolution of a Gaussian instrument response function (IRF) with a product of a step function with bi-exponential decay was used as a fit model. The step function describes the drop of reflectivity due to the XUV photon absorption followed by fast Auger decay and e–e scattering, which in turn changes the electron and hole density in the conduction and valence band, correspondingly. These hot electrons then thermalize via different processes (e. g., further e–e scattering, e-phonon coupling, e-diffusion into the bulk), which can be described by a “fast” and a “slow” exponential component of the reflectivity recovery. For more details, see Gahl *et al.*, 2008, Beye *et al.*, 2012, Eckert *et al.*, 2015 and references therein. Note, that this measured time response of the TRIXS spectrometer is not the ultimate possible time resolution of the end-station, but was measured for particular FEL and PG monochromator settings determined by the needs of a user experiment.

The employed technique demonstrated excellent sensitivity, proving that a reflectivity change of less than 0.06% can be confidently identified in short time. Furthermore, the FEL flux at the sample is sufficient to perform these measurements even when the PG1 primary monochromator is operated in the first diffraction order.

The ultimate time resolution of the TRIXS end-station can be estimated from the minimal values of all factors in σ_IRF_. The effective FEL pulse duration σ_FEL_ at the sample depends on the chosen dispersion/energy resolution of the primary PG beamline monochromator (Wellhöfer *et al.*, 2007, Gerasimova *et al.*, 2011). In the high-resolution mode, the monochromator diffraction grating introduces a strong pulse front tilt, leading to a substantial longitudinal pulse elongation (potentially up to a picosecond), which adds to the initial FEL pulse duration. Thus, a compromise in terms of energy resolution is indispensable for the highest time resolution. The FEL pulse duration before the monochromator grating can be estimated with the help of a transverse deflecting microwave structure (TDS) located in the FLASH accelerator section (Altenmueller *et al.*, 1964, Röhrs *et al.*, 2009). The TDS measures the longitudinal electron bunch profile before the PG monochromator from which the photon pulse duration can be deduced (Düsterer *et al.*, 2014). Thus, the estimated and currently available ultimate time resolution of TRIXS, based on all the above given values, is approximately 170 fs FWHM.

### G. Software control

The experimental control at TRIXS is currently distributed over different software packages. The spectrometer itself is operated via a LabView based server and a client implemented by BESTEC GmbH, while the spectrometer CCD detector is integrated into the FLASH Distributed Object-Oriented Control System Framework (DOOCS) (Hensler and Rehlich, 1996). A Python-based (Python Software Foundation) software package provides measurements of the spectrometer dispersion and resolution. Moreover, integrated RIXS spectra can be easily plotted in the live mode. The RIXS spectra are stored in the FLASH DAQ system in a binary format and can be automatically converted into the “hdf5” format (HDF Group) for near-online analysis. Typical RIXS spectra are collected within approximately 30 min, while the online conversion into the hdf5 format takes approximately 5 min.

## III. PERFORMANCE RESULTS

### A. Resolving power

**FIG. 6.**
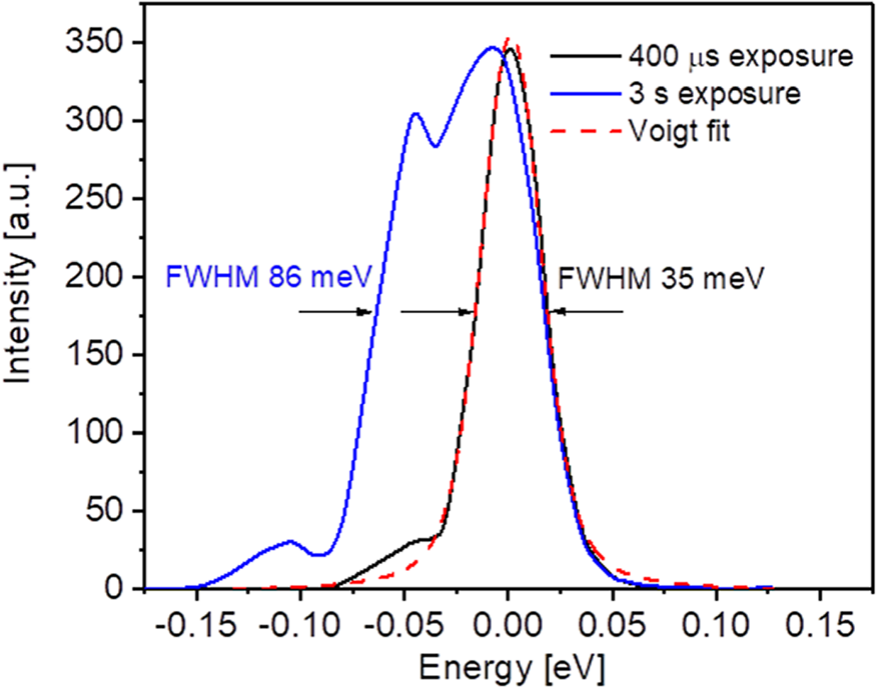
The spectrometer response function measured in the first diffraction order at 68 eV with exposure times of 400 μs (black) and 3 s (blue). The PG exit slit width was 50 *μ*m, corresponding to a bandwidth of approximately 10 meV. The Akima spline algorithm has been applied to the experimental data before fitting them with a Voigt function.

The spectral resolution of the RIXS spectrometer depends mainly on the energy bandwidth of the primary monochromator as well as on the FEL focal spot size on the sample. As was laid out before, in order to measure the weak RIXS signal, we accumulate for several seconds, thus averaging over all energy and pointing fluctuations caused by both the FEL lasing process and the mechanical instabilities of the beamline optical elements. This results in a broadening of the effective focus size on the sample and a larger effective bandwidth. In consequence, the effective resolving power of the spectrometer is reduced, as demonstrated in Fig. 6. Here, the FWHM of the elastic line is taken as the spectrometer instrument response function (IRF). Increasing the detector exposure time above a few hundreds of milliseconds, i.e., integrating over several FEL burst pulses broadens the elastic linewidth by a factor of almost 2.5.

**FIG. 7.**
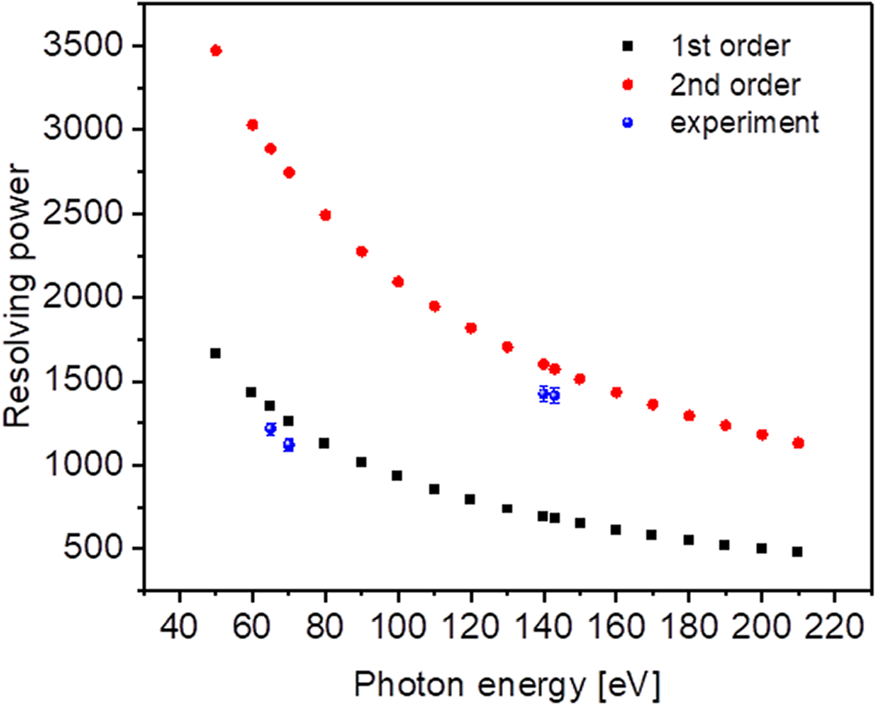
Spectrometer resolving power with 576 l/mm grating in first (black squares) and second (red circles) diffraction orders; measurement data are shown in blue.

Figure 7 depicts the calculated resolving power over the whole spectral range and measurements of the first spectrometer stage utilizing the 576 l/mm grating both in first and second diffraction orders. The primary PG1 monochromator used an exit slit width of 50 *μ*m, resulting in an energy bandwidth on the sample, corresponding to 0.01 – 0.03% of the photon energy (primary resolving power of 3000–10 000).

**FIG. 8.**
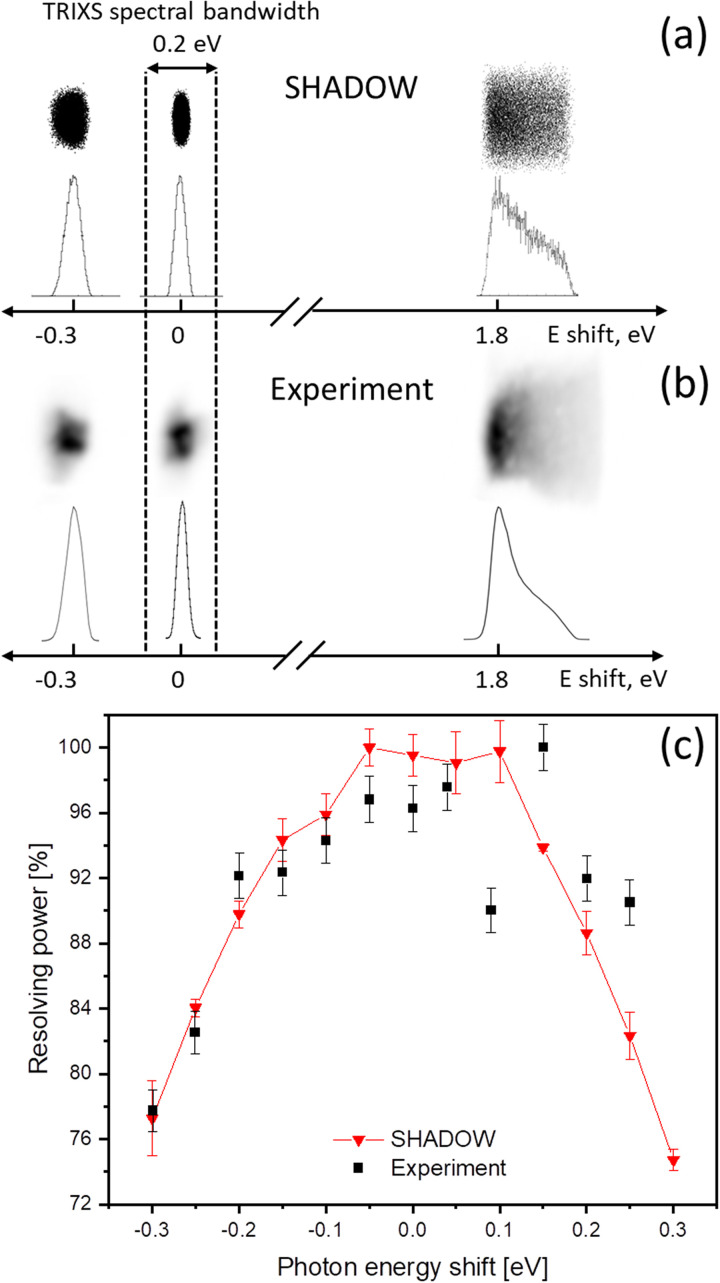
Distortion of the elastic line when tuning the PG1 monochromator far from the set photon energy of the TRIXS spectrometer estimated with SHADOW (a) and measured (b). The resulting decrease in the resolving power is shown in (c). The spectrometer high-resolution bandwidth is shown with dashed vertical lines. It corresponds to a resolution decrease of less than 8% compared to the possible maximum for the given photon energy of 68 eV. Error bars are standard deviations, which for SHADOW simulations were calculated by using different seeds in the Monte Carlo algorithm.

The Czerny–Turner optical scheme of the spectrometer results in a narrow spectral bandwidth with a high resolution. Beyond this region, the resolution rapidly decreases due to dominating coma aberrations. This is demonstrated by simulations performed in XOP SHADOW (Cerrina and Sanches del Rio, 2009) and validated by measurements of the elastic line broadening and shape distortion as a function of photon energy detuned from the spectrometer nominal central energy setting (Fig. 8). For this study, the angle of incidence of the spectrometer grating (pitch) was set to match the FEL photon energy selected by the PG1 beamline monochromator and its exit slit. By operating the beamline monochromator in a high-resolution mode, this setting corresponds to the most narrow and symmetric elastic line and thus to the best spectrometer resolution [see the elastic line in the 0.2 eV area in Figs. 8(a) and 8(b)]. Then, the PG1 monochromator energy was scanned across the FEL bandwidth (typically 1% of the fundamental photon energy), keeping the spectrometer grating fixed at the initial photon energy. When scanning the monochromator, the mismatch between the FEL photon energy and the analyzing spectrometer energy—determined by the grating pitch—increases, which leads to a constantly increasing beam path deviation of the dispersed light after the spectrometer grating from the optical axis of the refocusing off-axis parabola OAP2. This discrepancy, in turn, causes coma aberrations, which manifest themselves in a distorted and expanded elastic line shape and, therefore, in a reduced energy resolution of the spectrometer [Fig. 8(c)].

In order to reproduce the given experimental conditions, the simulations were performed for a photon source with 68 eV energy, uniform energy distribution, and 40 meV bandwidth; a source size of H × V = 40 × 25 *μ*m^2^ (ellipse, FWHM) and a uniform angular distribution of H × V = 0.088 × 0.038 rad^2^. The spectrometer grating angle (576 l/mm line density) was set to focus on the photon energy of 68 eV and the simulations included slope errors for all optical elements calculated via the “waviness” pre-processor included in SHADOW (Cerrina and Sanches del Rio, 2009). Slope errors are 4.85 *μ*rad (rms) and 0.485 *μ*rad (rms) for OAP mirrors and gratings, respectively.

The resolving power of the spectrometer decreases already by 8% above a bandwidth of 200–250 meV, which makes it necessary to scan the spectrometer energy to observe spectral changes over a broader energy range, if high-resolution information is required in a large spectral band.

### B. Throughput

**FIG. 9.**
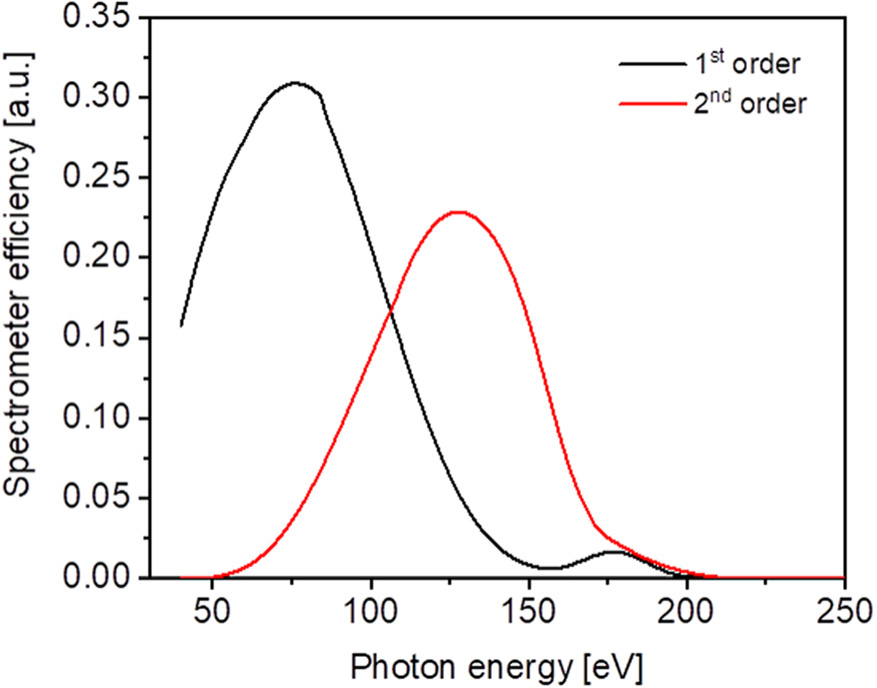
Combined efficiency of the first stage of the TRIXS spectrometer operating in the first (black) and second (red) diffraction order. The sensitivity of the CCD chip is not included.

The first stage spectrometer throughput is calculated to be on the order of 25–30% as shown in Fig. 9. Estimations were done by combining the measured 576 l/mm grating efficiency Dziarzhytski *et al.* (2018) with the reflectivity of the DLC coated mirrors calculated in SHADOW. The sensitivity of the CCD chip was not taken into account.

### C. Static RIXS measurements

**FIG. 10.**
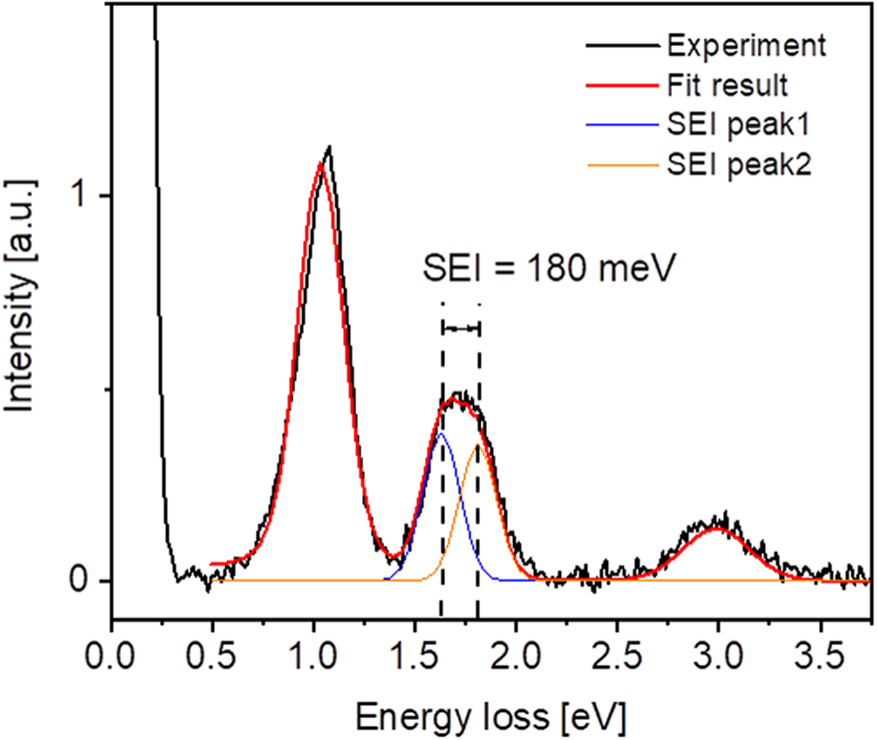
Static RIXS spectrum of NiO excited at the Ni M-edge at 70.1 eV. The primary PG1 monochromator bandwidth was set to 15 meV. The splitting of 180 meV of the d-d-excitation peak at 1.7 eV is caused by the superexchange interaction (SEI) (Chiuzbăian *et al.*, 2005). The elastic line is cut off for better presentation of the inelastic scattering peaks.

To demonstrate the TRIXS spectrometer performance, we present static RIXS measurements of NiO which was resonantly excited by the FEL at the Ni M_2,3_-edge at 70.1 eV photon energy (see Fig. 10).

For this measurement, FLASH was operated in burst mode with 4000 pulses/second. The primary PG monochromator was set to 15 meV bandwidth. The measured TRIXS resolution was 86 meV. Figure 10 shows the elastic line (intensity is cut in the graphical presentation) and the characteristic *d-d* excitations at 1.05 eV, 1.7 eV, and 3.3 eV which agree very well with the data recorded at a synchrotron (Chiuzbăian *et al.*, 2005). The central energy of the spectrometer was set to the 1.7 eV energy loss feature. The width of the sub-peaks at 1.7 eV energy and the distance between them agrees well with the magnetic excitation of NiO described in (Chiuzbăian *et al.*, 2005). The spectrum was recorded as an averaged sum of multiple background-corrected images each having 7 s exposure time. The total acquisition time of the spectrum was 30 min.

### D. Time-resolved RIXS measurement

**FIG. 11.**
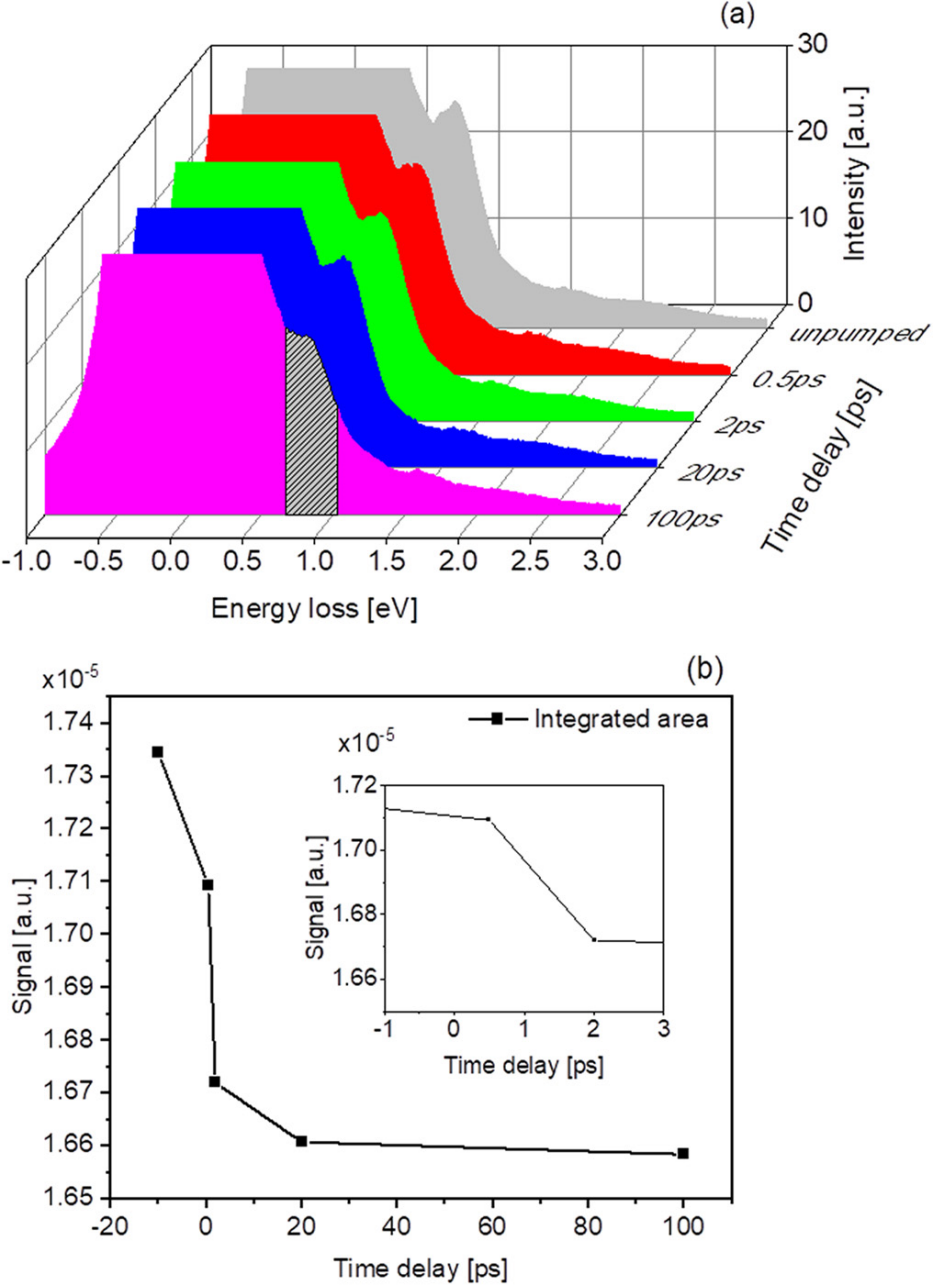
The time-resolved Co M-edge RIXS spectrum of cobalt oxide pumped at 400 nm (a), and the evolution of the d-d excitation peak integrated over the shaded area (b). The inset in (b) demonstrates the sub-picosecond dynamics of the d-d excitation upon optical pumping.

Time-resolved RIXS measurements have been successfully performed by FLASH users already on different systems utilizing all available optical wavelengths. Some of the co-authors of this work (S.D., P.S.M., R.Y.E., J.O.S., G.B., and M.B.) have worked on time-resolved Co M-edge RIXS on cobalt oxide and preliminary results of this research project are presented here. Figure 11(a) shows the Co M-edge RIXS spectra of cobalt oxide pumped with 400 nm optical laser light and FEL-probed at 65 eV at different time delays. It was possible to trace a sub-picosecond dynamics of the d-d excitations of the Co ion at around 0.9 eV, and its evolution was followed down to 100 ps [see Fig. 11(b)]. The measured TRIXS spectrometer resolution was 65 meV. The sample was kept at room temperature.

The sensitivity to changes in experiments with the TRIXS end-station depends strongly on the overall signal strength from the sample. As a very rough estimate, based on the measurements we have already performed on various materials, we can state that we are typically able to observe changes between 0.05% and 0.5% after 30 minutes of RIXS measurement with FLASH operating at an average 4 kHz repetition rate. Thus, such experiments in the soft X-ray range are possible only with high repetition rate FELs like FLASH.

## IV. CONCLUSION

The feasibility of soft x-ray time-resolved RIXS on solid samples with a femtosecond time resolution has been demonstrated at FLASH utilizing the new end-station TRIXS, combining a high-resolution RIXS spectrometer with a high-repetition rate FEL and a synchronized optical laser system in a pump-probe scheme. A summary of all important TRIXS parameters is given in Table III. The high energy resolution of the instrument, its relatively large acceptance angle and efficiency, as well as sub-picosecond time resolution clearly make dynamical studies of, e.g., 3d transition metals and 4f rare earth elements possible. Spatial and temporal overlap between FEL and optical laser can be efficiently established down to 10 *μ*m and to the femtosecond level, respectively, using the diagnostics at the end-station. The high number of FLASH photon pulses (up to 5000 pulses/second) in combination with the high flux at the sample and optimized spectrometer throughput permits the recording of photon-hungry highly resolved RIXS spectra with sufficient signal levels to enable time-resolved studies within reasonable measurement time.

**TABLE III.** Summary of the parameters of the TRIXS end-station.

	TRIXS	
Spectral range, eV	36–210	
Spectral resolution, meV	35–160	
Resolving power E/dE	500–3500	
Time resolution FWHM, fs	170–300^a^	
Acceptance angle H x V, mrad^2^	82.6 × 37	
Throughput	0.05–0.3	
	FEL	Optical laser
Pulse energy at the sample, *μ*J	≤2	≤ 1.5 (267 nm); ≤ 10 (400 nm); ≤ 25 (800 nm);
N_photons_ on the sample per pulse	≤10^10^	≤ 10^12^
Focal spot size on the sample H x V, *μ*m^2^	12 × 6^b^	100 × 50 (267 nm); 160 × 80 (400 nm); 200 × 100 (800 nm)
Pulse duration, ps	0.05–1.5^c^	0.09–1.5^c^

^a^ Depends on the chosen energy resolution.

^b^ Vertical FEL spot size on sample dependency is w/5, with w being the PG monochromator exit slit width.

^c^ Depends on experimental requirement.

We should note that in 2021, a new FLASH pump-probe femtosecond laser will provide 1030 nm photons in the fundamental wavelength with pulse energies of up to 70 *μ*J per pulse, as well as the second, third, and fourth harmonics with a pulse duration below 100 fs. Moreover, a major upgrade of the FLASH facility within the FLASH2020+ project [see the conceptual design report in (Beye *et al.*, 2020)] is planned, including variable gap/polarization undulators for FLASH1 as well as seeding. A high-repetition rate seeded FLASH will provide fully coherent and Fourier-limited photon pulses, which in turn will increase the XUV fluence on the sample as well as its intensity stability. Furthermore, seeding will also decrease the timing jitter between FEL and optical laser pulses as well as the FEL intra-train jitter, and last but not least, the pointing stability of the FEL beam. Altogether, this will significantly improve the TRIXS time and energy resolution as well as the signal-to-noise ratio of the transient RIXS signals within a shorter measurement time.

## AUTHORS' CONTRIBUTIONS

M.R. made the conceptual design and built the spectrometer within the BMBF project.

S.D., M.Bi., B.D., H.W., M.R., and G.B. commissioned the spectrometer at FLASH.

F.S. performed metrology on the optical elements.

S.D. and G.B. initiated the time-resolved RIXS project and together with H.R., H.W., and A.S. designed, built, and commissioned the laser infrastructure with support from W.W. and the FLASH facility. S.D., A.W., P.S.M., R.Y.E., J.O.S., H.R., M.S., H.W., B.G.-L., S.G.C., W.W., M.Be., M.R., and G.B. contributed equally to the commissioning of the TRIXS endstation.

M.Be. helped to implement the time zero diagnostics.

R.Y.E., J.O.S., C.B., and M.Be. contributed to the software development.

S.D., A.W., P.S.M., R.Y.E., J.O.S., H.R., H.W., M.S., A.S., B.G.-L., S.G.C., W.W., M.Be., MR, and GB performed the RIXS experiment on NiO.

P.S.M. was a principal investigator on the tr-RIXS studies of cobalt oxide compounds and performed the experiment together with S.D., P.S.M., R.Y.E., J.O.S., M.Be., and G.B.

S.D. and G.B. wrote the manuscript with contributions from M.Be., S.G.C., P.S.M., and M.R. The whole manuscript was further discussed with all co-authors.

## Data Availability

Raw data were generated at the FLASH (DESY) large scale facility. Derived data supporting the findings of this study are available from the corresponding author upon reasonable request.
